# Protein Regulator of Cytokinesis 1 (PRC1) Upregulation Promotes Immune Suppression in Liver Hepatocellular Carcinoma

**DOI:** 10.1155/2022/7073472

**Published:** 2022-08-09

**Authors:** Canjing Zhang, Huiwen Xu, Xianxian Sui, Ting Wu, Bobin Chen, Songmei Wang, Xuanyi Wang

**Affiliations:** ^1^Institutes of Biomedical Sciences, And Key Laboratory of Medical Molecular Virology of Ministry of Education & Ministry of Health, School of Basic Medical Sciences, Fudan University, 200032 Shanghai, China; ^2^Laboratory of Medical Molecular Biology, Experimental Teaching Center, School of Basic Medical Sciences, Fudan University, 200032 Shanghai, China; ^3^Department of Hematology, Huashan Hospital, Fudan University, 200040 Shanghai, China

## Abstract

Liver hepatocellular carcinoma (LIHC) is a malignant cancer with widespread prevalence. The suppressive immune environment causes largely refractory to current treatment. The protein regulator of cytokinesis 1 (PRC1) is an essential gene for cytokinesis and is involved in cancer pathogenesis. However, the functions of PRC1 have been barely clarified, especially in LIHC. Here, we investigated the expression, prognostic value, and functions of PRC1 in LIHC. Pan-cancer analysis revealed the overexpression of PRC1 in the Cancer Genome Atlas (TCGA) database. Four LIHC datasets from the Gene Expression Omnibus (GEO) database confirmed the PRC1 overexpression in LIHC. The mRNA and protein levels of PRC1 in LIHC cells were higher than in normal liver cells. The overexpression of PRC1 predicted progressed clinical stage and poor prognosis of LIHC. We further investigated the functions of PRC1 by performing the Gene Ontology (GO), Kyoto Encyclopedia of Genes and Genomes (KEGG) analyses, and Gene Set Enrichment Analysis (GSEA) of its coexpressing genes. High PRC1 expression was associated with increased genome instability of LIHC. Moreover, PRC1 was positively correlated with the infiltration of suppressive immune cells like T regulatory cells (Tregs) and polymorphonuclear myeloid-derived suppressor cells (PMN-MDSCs) and was negatively correlated with the effector immune cells' infiltration, including B cells and CD8+ T cells. In addition, PRC1 was positively correlated with the expression of tumor immune checkpoint molecules. Taken together, PRC1 overexpression contributes to the genome instability and the suppressive immune microenvironment of LIHC. Thus, PRC1 has the potential to be a prognostic marker and therapeutic target of LIHC.

## 1. Introduction

Liver hepatocellular carcinoma (LIHC) accounts for 90% of primary liver cancer with an incidence of 850,000 newly diagnosed cases every year. Quick and internal development often causes the delay of diagnosis, making LIHC the leading cause of cancer-related deaths. LIHC is resistant to most current chemotherapies, which have shown no survival benefits to advanced LIHC patients [1]. Immune checkpoint inhibitors (ICIs) have improved the survival of LIHC patients, even though its introduction to LIHC was behind other tumors. Inhibitors of programmed cell death/-ligand 1 (PD-1/PD-L1) have shown revolutionary antitumor activity in a subset of advanced patients [2]. However, problems still exist with ICIs in their antitumor efficacy and adverse events. Single agent is limited in efficacy: the ORR and median OS of anti-PD-1 ICI were 15% and 1 year, respectively, in patients previously treated with sorafenib [3, 4]; monotherapy of anti-PD-1 ICI showed no benefit for OS as first-line (nivolumab vs sorafenib) and second-line (pembrolizumab vs placebo) treatment [5, 6]. The combination of ICIs increases the response rate and leads to a promising OS, but at the cost of increased toxicity [7, 8]. The hepatic events incidence of ICIs is slightly higher in LIHC than in other cancers. Moreover, no reliable markers are available for immunotherapy guidance currently. The immune-evading mechanism of LIHC needs to be further investigated, and new treatment targets and reliable markers are under exploring.

To evade the antitumor immunity, LIHC cells evolve to less immunogenic phenotypes with reduced expression of cancer antigens and MHC molecules and increased expression of immune checkpoint ligands. Altered tumor microenvironment (TME) also enables LIHC progression [9]. TME is a complex of tumor cells, immune cells, hepatic cells, and fibroblasts, along with soluble factors like cytokines. The complicated interaction in TME of LIHC results to immune evasion. Both of the adaptive and innate immune systems are damaged in TME of LIHC, due to the expression of inhibitory receptors, and the infiltration of immune-suppressive cells like myeloid-derived suppressor cells (MDSCs), tumor-associated macrophages (TAMs), and T regulatory cells (Tregs) [10]. These suppressive immune cells are abundant in TME of LIHC and cooperating to generate an immunosuppressive environment. Tregs are a subset of immunosuppressive T cells identified as CD4+ CD25+ (the *α*-chain of IL-2 receptor) and characterized by Forkhead box protein P3 (FoxP3) expression [11, 12]. Treg cells contribute to cancer development and progression by suppressing T effector cell (Teff) functions and inducing the overexpression of immunosuppressive molecules [13]. Tregs highly express PD-1, which leads to the tumor resistance to PD-1/PD-L1 blockade treatment [14]. Increased intratumoral Tregs inhibit the proliferation and activation of CD8+ and CD4+ T cells and result in the production of transforming growth factor-*β* (TGF-*β*) and interleukin-10 (IL-10), which in turn favor the survival and expansion of Tregs [15]. The high density of Treg cells in TME of LIHC correlates with poor prognosis [16]. A specific subset of MDSCs can induce CD4+ T cells to differentiate into Tregs [17]. Moreover, MDSCs support tumor progression by producing vascular endothelial growth factor (VEGF), which promotes vascularization and angiogenesis of the malignant tissue [18]. Beside cells, the soluble molecules should not be underestimated. They also contribute to the poor prognosis of LIHC and determine the patients' response to sorafenib and pembrolizumab, which are currently the first-line therapies for LIHC [19, 20]. TGF-*β*, IL-10, and VEGF impair the functions of T cells and NK cells, induce the generation of Tregs, and downregulate the T cell stimulatory capacity of antigen presenting cells (APCs) [21–25].

Effector lymphocytes express immune checkpoints molecules to prevent immune overactivation. LIHC utilizes this mechanism to evade immune responses by expressing the inhibitory checkpoint ligands [26]. Inhibitory ligands and receptors include PD-1/PDL-1, and cytotoxic T lymphocyte-associated antigen 4 (CTLA4), T cell immunoglobulin and mucin domain containing-3 (TIM3), and lymphocyte-activation gene 3 (LAG3) [27]. ICIs are monoclonal antibodies that block the interaction of checkpoint proteins with their ligands, thereby preventing the inactivation of T cells. However, the blockade of checkpoint molecules can lead to frequent incidence of immune-mediated adverse events (IMAEs), since they are essential for immune homeostasis [28]. Furthermore, suitable biomarkers to guide the development and monitor the management of ICIs are lacking in LIHC. For example, LIHC patients showed a similar response to ipilimumab and nivolumab treatment, irrespective of expression of PD-L1 [29]. This indicates that the current biomarkers may be incapable to inform clinical decisions. Thus, massive data analyses are needed to identify better prognosis biomarkers and immunotherapy targets.

The protein regulator of cytokinesis 1 (PRC1) regulates parallel microtubule polarizing and contractile ring assembly. It is an essential factor for cytokinesis and cell cleavage. Recent studies found that PRC1 might play an important role in tumorigenesis [30]. The deregulation of PRC1 promoted chromosomal instability (CIN) [31]. By far, PRC1 has been fond upregulated and correlated with poor prognosis in colon, breast, liver, and prostate cancers and oral squamous cell carcinoma [32–35]. However, the underlying molecular mechanisms concerning cancer development and fate remain poorly understood. Previous studies reported PRC1 as a substrate of cyclin-dependent kinases (CDKs) and polo-like kinase 1 (PLK1), which are essential cell cycle regulators [36]. In LIHC cells, PRC1 can be activated by Wnt signaling pathway and vice versa [30]. The function of PRC1 in LIHC may also be reliant on p53 [37]. Given the essential role of PRC1 in LIHC growth, we seek to understand the comprehensive function of PRC1. Here, we utilize the bioinformatics methods to investigate the possible molecules, signaling pathways regulated by PRC1, and the biological process PRC1 might involve, seeking to identify new biomarkers for LIHC prognosis and targets for clinical treatment.

## 2. Materials and Methods

### 2.1. PRC1 Expression Profiling

The RNA sequencing data of LIHC patients and healthy liver tissues were acquired from the GEO database (GSE60502, GSE84402, GSE84598, and GSE112790) [38–41]. The differentially expressed genes (DEGs) of each dataset were analyzed by R Limma package. The pan-cancer analyses of PRC1 expression were also conducted by R software, using data acquired from the TCGA database through the Genomic Data Commons data portal (GDC, https://portal.gdc.cancer.gov/) containing 33 kinds of cancers, including 374 LIHC cases, while the normal samples were from both the TCGA and GTEx database[42]. Immunohistochemistry (IHC) staining images of 16 LIHC and 13 normal liver tissues were extracted from the Human Protein Atlas (HPA, http://www.proteinatlas.org) [43]. UALCAN (http://ualcan.path.uab.edu/) database showed the PRC1 expression of LIHC in different clinical stages or in different TP53 mutation status [44].

### 2.2. Cell Culture, RNA Isolation, and Quantitative Real-Time PCR (qRT-PCR)

The human LIHC cell line SNU449 and normal liver cell line QSG-7701 were cultured in Dulbecco's modified Eagle's medium (DMEM, Gibco, USA) containing 10% fetal bovine serum (FBS, BI, USA) and in an incubator with 5% CO2 at 37°C. RNA isolation was conducted using TRIZOL (Life Technology) agent according to the manufacture's protocol. The complementary DNA (cDNA) was reversely transcribed using the ReverTra Ace qPCR RT Master Mix (TOYOBO, Japan). qRT-PCR was performed in triplicate using SYBR green real-time PCR master mix (TOYOBO, Japan). Primers of PRC1 (forward, 5′-ACAGACAGAGACAGAGATG-3′; reverse, 5′- GCCGAATGCTACTATTGG-3′) and *β*-actin (forward 5′-GAAGATCAAGATCATTGCTCCT-3′; reverse, 5′-TACTCCTGCTTGCTGATCCA-3′) were used.

### 2.3. Cell Lysis and Western Blot

QSG7701 and SNU449 cells were collected and lysed in RIPA buffer (Beyotime, China) supplemented with PhosSTOP (Roche, USA) and 1 mM PMSF (Beyotime, China). Proteins were mixed with SDS loading buffer (Beyotime, China) and boiled for 5 min. Then, the proteins were loaded into 4-12% SDS PAGE gels (Genescript, China) and subjected to electrophoresis, after which the proteins were transferred to PVDF membranes and went through antibody incubation. Primary antibodies used were as follows: PRC1 (#6290001, Biolegend, USA) and GAPDH (#2118, CST, USA). GAPDH was used as a loading control.

### 2.4. Survival Analysis

The overall survival (OS), progression-free interval (PFI), and disease-specific survival (DSS) analyses of LIHC patients with high or low PRC1 expression in the TCGA database were conducted by survival package and survminer package in R software. Also, we used KM Plotter (http://kmplot.com) website tool to analyze whether the prognostic value of PRC1 was associated with Treg, macrophage, or B cells' infiltration in LIHC [45]. A total of 374 LIHC samples from TCGA database were stratified into two groups, Treg enriched or decreased. Then, we analyzed the OS and recurrence-free survival (RFS) of high and low PRC1 cases separated by the median expression in each group. We acquired the survival curve according to PRC1 expression of HCCDB 18 dataset containing 212 LIHC tumor tissues and 177 adjacent tissues from the HCCDB (http://lifeome.net/database/hccdb/home.html) database to validate the prognostic values of PRC1 in LIHC. To estimate the diagnostic value of PRC1, we created a nomogram using rms package in R software and performed the ROC analysis using the pROC package in R software [46].

### 2.5. Gene Ontology (GO) Term and Kyoto Encyclopedia of Genes and Genomes (KEGG) Pathway Enrichment Analysis and Gene Set Enrichment Analysis (GSEA)

We performed the Pearson's correlation analysis to acquire the coexpression genes of PRC1 using RNA sequencing data of LIHC in the TCGA database [47]. The top 300 genes were extracted and underwent GO analyses including molecular function (MF), cellular components (CC), biological processes (BP), and KEGG pathway analysis. GSEA analysis of the top 300 coexpression genes of PRC1 was conducted with GSEA software (V4.1.0).

### 2.6. Association of PRC1 with Somatic Mutations and Genome Instability in LIHC

The information of somatic mutations of LIHC cases were downloaded from TCGA database and analyzed by comparing cohorts separated by median PRC1 expression. A total of 369 samples with mutations were detected, of which the mapping samples contained 242 (65.6%). We used chi-square test to evaluate the difference of gene mutation frequency in each group of samples. The simple nucleotide variation dataset of all TCGA samples processed by MuTect2 software was downloaded from GDC [48]. We calculated the tumor mutation burden (TMB) of each tumor using the TMB function of the maftools package in R software. The microsatellite instability (MSI) score of each tumor was acquired from the previous study [49]. The homologous recombination deficiency (HRD) data and the loss of heterozygosity (LOH) data were obtained from the previous studies [50]. Spearman correlation analysis was conducted to investigate the correlation of PRC1 expression with the above factors.

### 2.7. Immune Cells' Infiltration and Gene Correlation Analyses

We analyzed the correlation between PRC1 expression sequenced in bulk LIHC tumor tissues and their infiltration abundance of Treg and MDSC cells in the TCGA database by CIBERSORT [51], CIBERSORT-ABS [52], QUANTISEQ [53], and TIDE [54] algorithms using the website tool of Tumor Immune Estimation Resource (TIMER, https://cistrome.shinyapps.io/timer/) [55].The relationship between PRC1 expression and the gene markers of Treg and MDSC was plotted by TIMER. The association between PRC1 expression and the infiltration of Teff cells in LIHC of TCGA database was analyzed by EPIC algorithm [56]. The correlation between expression of PRC1 and eight immune checkpoint genes was plotted by R ggstatsplot package.

### 2.8. Prognostic Value of the Immune-Related Gene Set Signature

According to the GSEA analysis results, the immune-related gene subset was extracted from the PRC1 coexpressing genes. The prognostic value of these genes was tested as signatures. Time ROC analysis was conducted by R timeROC package to investigate the predictive accuracy of the genes and risk score. The least absolute shrinkage and selection operator (LASSO) regression algorithm was performed using 10-fold cross-validation [57]. The OS and progression-free survival (PFS) data were acquired from the TCGA database [58] and were analyzed to compare the survival difference between groups, with the *p* values and hazard ratio with 95% confidence interval (CI) generated by the log-rank tests and univariate Cox proportional hazards regression.

### 2.9. Statistical Analysis

Data were expressed as mean ± SD. Analyses were performed in GraphPad Prism (version 8.0) or R software (v3.4.4). Comparisons of the PRC1 expression between LIHC and normal controls were performed using Student's *t*-test or chi-square test. Gene expression correlations were evaluated by Spearman's correlation. *p* < 0.05 was considered statistically significant.

## 3. Results

### 3.1. The Pan-Cancer Upregulation of PRC1

**Figure 1:**
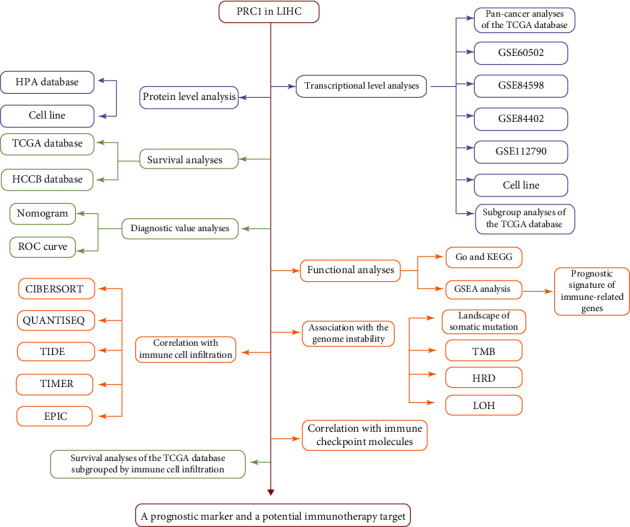
Study design and method implementation in this work.

**Figure 2:**
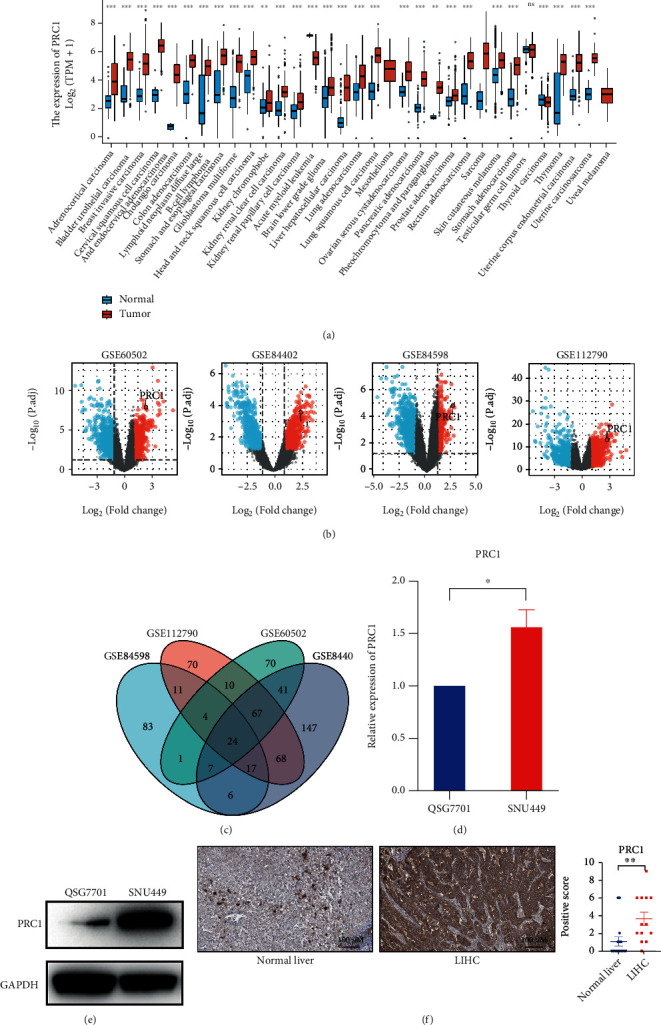
The expression of PRC1 was elevated in LIHC. (a) The pan-cancer analysis of PRC1 expression. (b) The volcano map of differentially expressed genes (DEGs) between LIHC and normal liver, obtained from four GEO datasets: GSE60502, GSE84402, GSE84598, and GSE112790. PRC1 was noted. (c) The intersection of the DEGs from four GEO datasets. (d) The mRNA level of PRC1 in QSG7701 and SNU449 cells. (e) The protein level of PRC1 in QSG7701 and SNU449 cells. (f) The IHC results of PRC1 in LIHC and normal liver tissues from the HPA database. The scored results were as shown in the right panel. ns, *p* ≥ 0.05; ∗*p* < 0.05; ∗∗*p* < 0.01; ∗∗∗*p* < 0.001.

A schematic workflow diagram of our study is shown in Figure 1. We first accessed the pan-cancer expression of PRC1 in TCGA database. Pan-cancer analysis revealed the upregulation of PRC1 in adrenocortical carcinoma (ACC), bladder urothelial carcinoma (BLCA), breast invasive carcinoma (BRCA), cervical squamous cell carcinoma and endocervical adenocarcinoma (CESC), cholangiocarcinoma (CHOL), colon adenocarcinoma (COAD), lymphoid neoplasm diffuse large B cell lymphoma (DLBC), stomach and esophageal carcinoma (STES), glioblastoma multiforme (GBM), head and neck squamous cell carcinoma (HNSC), kidney chromophobe (KICH), kidney renal clear cell carcinoma (KIRC), kidney renal papillary cell carcinoma (KIRP), brain lower-grade glioma (LGG), LIHC, lung adenocarcinoma (LUAD), lung squamous cell carcinoma (LUSC), ovarian serous cystadenocarcinoma (OV), pancreatic adenocarcinoma (PADD, or PAAD), pheochromocytoma and paraganglioma (PCPG), prostate adenocarcinoma (PRAD), rectum adenocarcinoma (READ), sarcoma (SARC), skin cutaneous melanoma (SKCM), stomach adenocarcinoma (STAD), thyroid carcinoma (THCA), thymoma (THYM), uterine corpus endometrial carcinoma (UCEC), and uterine carcinosarcoma (UCS) (Figure 2(a)).Also, PRC1 was found overexpressed in four LIHC datasets in the GEO database (GSE60502, GSE84402, GSE84598, and GSE112790) compared with normal liver tissues (Figure 2(b)). The intersection of the four DEG sets consists of 24 genes, including PRC1 (Figure 2(c)). Subsequently, we conducted the qRT-PCR and Western Blot assays to detect the mRNA and protein levels of PRC1 in a LIHC cell line SNU-449 and a normal liver cell line QSG-7701. The mRNA and protein levels of PRC1 in SNU449 cells were significantly higher than in QSG-7701 cells (Figures 2(d) and 2(e)). Consistently, the protein level of PRC1 in LIHC patients' tissues was significantly increased compared with which in normal liver tissues (Figure 2(f)). These findings illustrated that PRC1 expression was upregulated in LIHC, indicating that PRC1 might play an important role in tumorigenesis.

### 3.2. High PRC1 Expression Is Associated with More Advanced LIHC Clinical Stages

**Figure 3:**
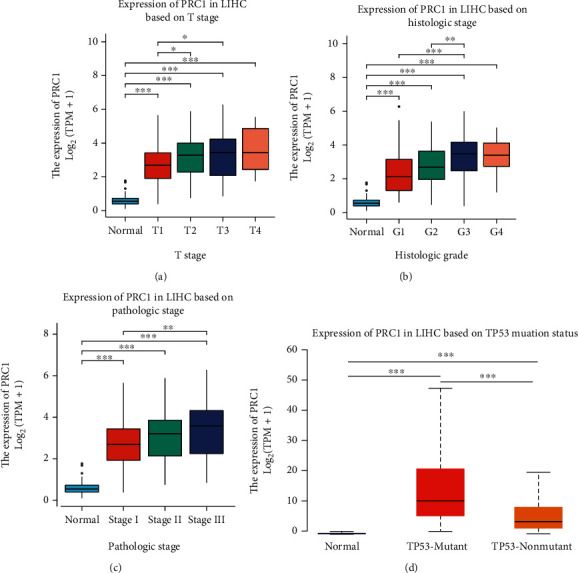
PRC1 expression among different groups of patients based on clinical parameters. Analyses are shown for T stage (a), histologic grade (b), pathologic stage (c), and TP53 mutant status (d). ∗*p* < 0.05; ∗∗*p* < 0.01; ∗∗∗*p* < 0.001.

The baseline characteristics of the LIHC patients from the TCGA database dichotomized by median PRC1 expression were described (Table 1). The baseline characteristics of the GEO dataset were listed in Supplementary Table 1. To better understand the correlation between PRC1 and LIHC progression, we investigated the PRC1 expression in LIHC patients grouped by different clinical parameters. According to the tumor T stage, upregulation of PRC1 was observed in T1, T2, T3, and T4 stage compared with normal tissue. The expression of PRC1 in stages T2 and T3 was significantly higher than in T1 (Figure 3(a)). Based on the histologic stage, PRC1 expression was higher in G1, G2, G3, and G4 stages than in normal group, while G3 stage was significantly higher than in G2 and G1 stage (Figure 3(b)). In terms of pathologic stage, the PRC1 level was significantly elevated in stages I, II, and III of LIHC tissues compared with normal tissues, and PRC1 expression in stage III was higher than in stage I (Figure 3(c)). TP53 mutation is a marker of tumor mutation burden (TMB). Upregulation of PRC1 expression was also observed in both TP53-mutant and TP53 wild-type LIHC patients compared with normal controls, with the TP53-mutant LIHC patients bearing higher PRC1 expression (Figure 3(d)). These results suggest that PRC1 is associated with the progression of LIHC.

### 3.3. Increased PRC1 Expression Predicts a Worse Prognosis

**Figure 4:**
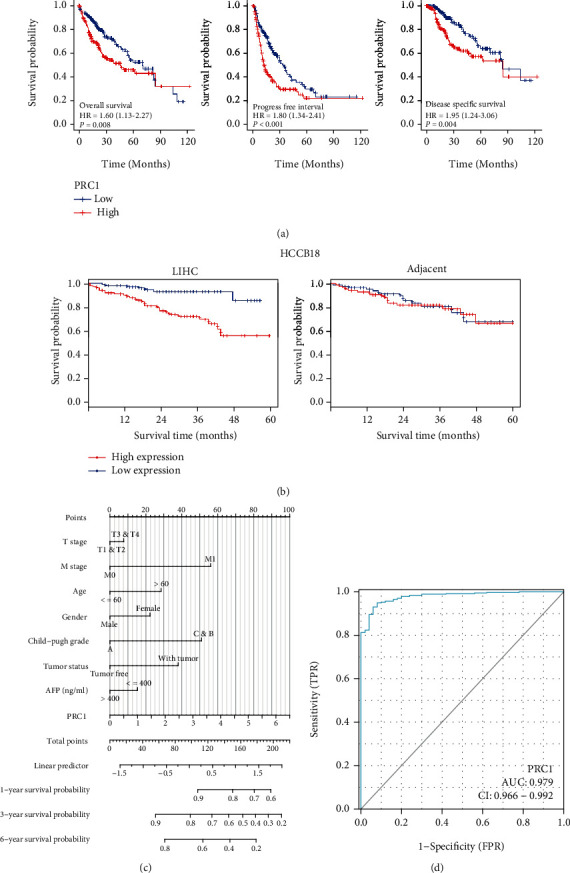
PRC1 upregulation was associated with worse prognosis. (a) The OS, PFI, and DSS survival curves of LIHC from the TCGA database. (b) OS survival curves of LIHC from HCCB18 in the HCCDB database. (c) Nomograms based on clinical factors and PRC1 expression. (d) ROC curve of PRC1. AUC was calculated and noted.

Given that PRC1 was associated with the development of LIHC, we examined the prognostic value of PRC1.By analyzing data in the TCGA database, we found that LIHC patients with higher expression of PRC1 exhibited worse outcomes of OS, PFI, and DSS (Figure 4(a)). For validation, survival curves of a LIHC dataset, HCCB18, were obtained from the HCCDB database. High PRC1 expression in LIHC tissues predicted worse OS, while in adjacent tissues did not (Figure 4(b)). A nomogram based on the Cox regression analysis was developed for prognostic prediction of PRC1 using the LIHC data from the TCGA database (Figure 4(c)). The nomogram showed the correlation of PRC1 expression with 1-, 3-, and 6-year survival probability, whereas higher expression of PRC1 predicted lower survival probabilities (Figure 4(c)). The ROC curve was plotted to measure the predictive performance of PRC1 prognostic risk model using the TCGA data (Figure 4(d)). The area under the curve (AUC) achieved 0.979 (Figure 4(d)). Other than LIHC, the pan-cancer prognostic value analyses of PRC1 showed that high PRC1 expression also predicted worse OS of glioma (GBMLGG), pan-kidney cohort (KICH+KIRC+KIRP, KIPAN), LGG, KIRP, mesothelioma (MESO), KIRC, ACC, KICH, LUAD, PAAD, acute myeloid leukemia (LAML), PRAD, SKCM, and CHOL, and also predicted worse PFI of GBMLGG, LGG, BRCA, LUAD, SARC, KIRP, KIPAN, PRAD, KIRC, BLCA, THCA, MESO, UVM, PAAD, PCPG, ACC, KICH, and CHOL (Supplementary Figure 1).

### 3.4. Identification of PRC1 Coexpressed Genes and Gene Ontology (GO) and Kyoto Encyclopedia of Genes and Genomes (KEGG) Pathway Analyses

**Figure 5:**
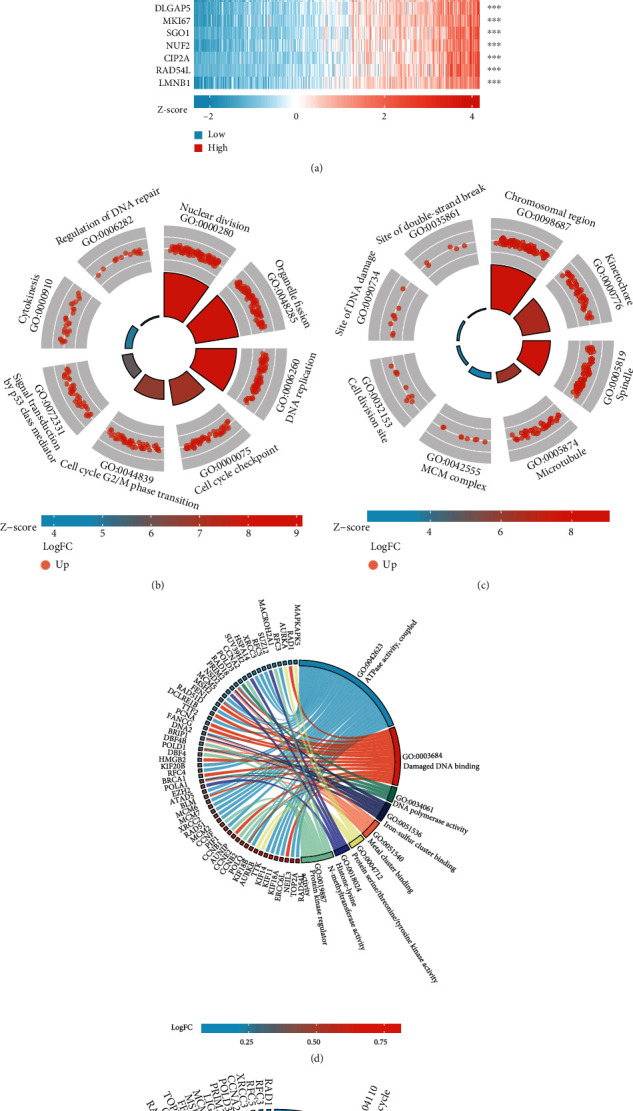
GO and KEGG enrichment analyses for genes co-expressed with PRC1. (a) Co-expression heat map of the TOP 50 genes related with PRC1. We conducted GO and KEGG analyses of the top 300 PRC1-related genes. Enrichment terms in BP (b), CC (c), MF (d), and KEGG pathway (e) were shown.

To understand the function of PRC1 comprehensively, we identified the genes coexpressed with PRC1 via LIHC dataset from the TCGA database. We extracted the top 300 genes related to PRC1 to explore its biological functions (Supplementary Table 2). The top 50 related genes were all positively correlated with PRC1 in LIHC and were shown by plot (Figure 5(a)). The top 300 related genes were used for KEGG and GO enrichment analyses. The representative BP, CC, MF, and KEGG pathways were presented (Figures 5(b)–5(e)). In terms of BP, PRC1 was related to cytokinesis, DNA replication, homologous recombination (HR), regulation of DNA repair, and cell cycle checkpoint and G2/M phase transition (Figure 5(b)). The CC that PRC1 involved included site of double-strand break, chromosomal region, and kinetochore (Figure 5(c)). The MF terms included ATPase activity, damaged DNA binding, polymerase activity, metal cluster binding, and protein kinase regulation (Figure 5(d)). KEGG analysis revealed the relationship between PRC1 and cell cycle, DNA replication, HR, Fanconi anemia (FA) pathway, cellular senescence, p53 pathway, and microRNAs (Figure 5(e)).

### 3.5. The Relationship between PRC1 and LIHC Genomic Instability

**Figure 6:**
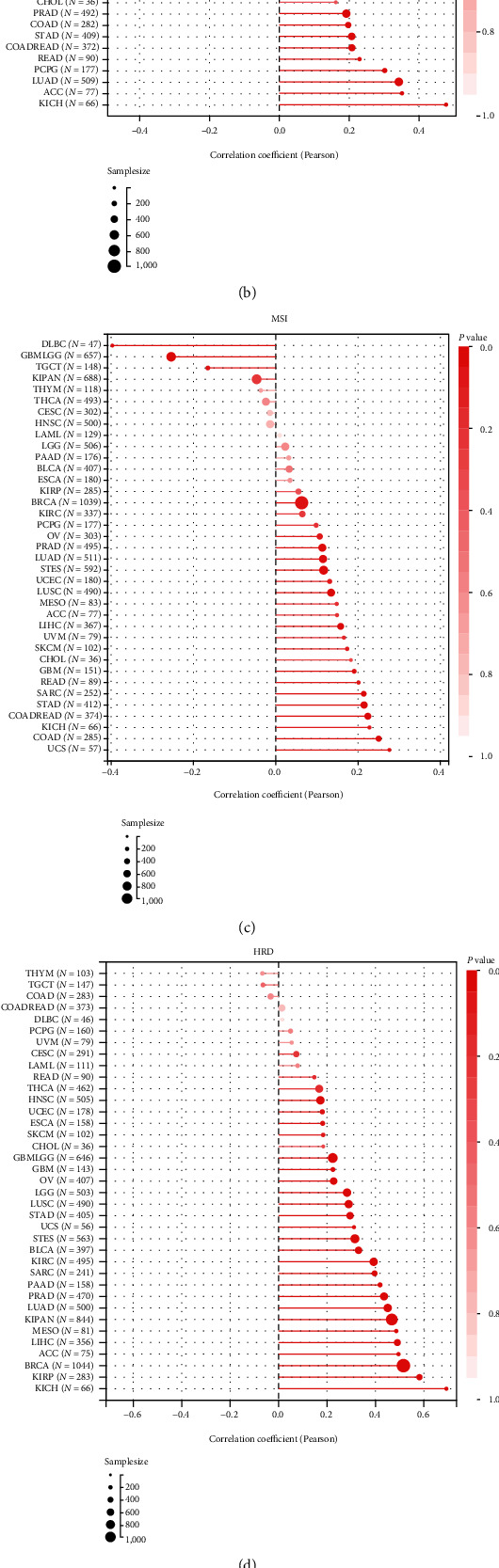
The relationship between PRC1 and genome instability of LIHC. (a) Landscape of somatic mutation in high and low PRC1 expression LIHC subpopulations. Genes are ranked by mutational frequency. Upper panel displays TMB score of each patient. (b–e) The relationship of PRC1 expression with TMB (b), MSI (c), HRD (d), and LOH (e).

Since PRC1 is correlated with HR and DNA damage repair (DDR) as revealed by GO and KEGG analyses, we further analyzed the relationship between PRC1 and the genomic instability of LIHC. We divided the LIHC patients in the TCGA database into two groups based on the median expression level of PRC1 and analyzed the mutation genes (Figure 6(a)). However, the genes with the top 15 mutation frequencies were the same in the high and low PRC1 subgroups, including TP53, CTNNB1, ALB, LRP1B, CACNA1E, RB1, NBEA, SPEG, TDRD5, IL6ST, FLG2, RBL2, ERICH3, KIAA1551, and ITGAD (Figure 6(a)).

TMB, MSI, HRD and LOH are biomarkers for predicting the tumor response to ICI therapy and/or chemotherapy [59–62]. PRC1 was found not related with TMB of LIHC. However, significant positive correlation of PRC1 with TMB was found in 15 kinds of tumors: GBMLGG, LUAD, COAD, READ, STES, KIPAN, STAD, PRAD, UCEC, KIRC, READ, PCPG, BLCA, ACC, and KICH (Figure 6(b)). Besides, PRC1 was positively correlated with the MSI of 12 types of cancers, the HRD of 24 types of cancer, and the LOH of 23 types of cancers, all including LIHC (Figures 6(c)–6(e)).

### 3.6. Gene Set Enrichment Analysis (GSEA) of PRC1 Coexpressed Genes

**Figure 7:**
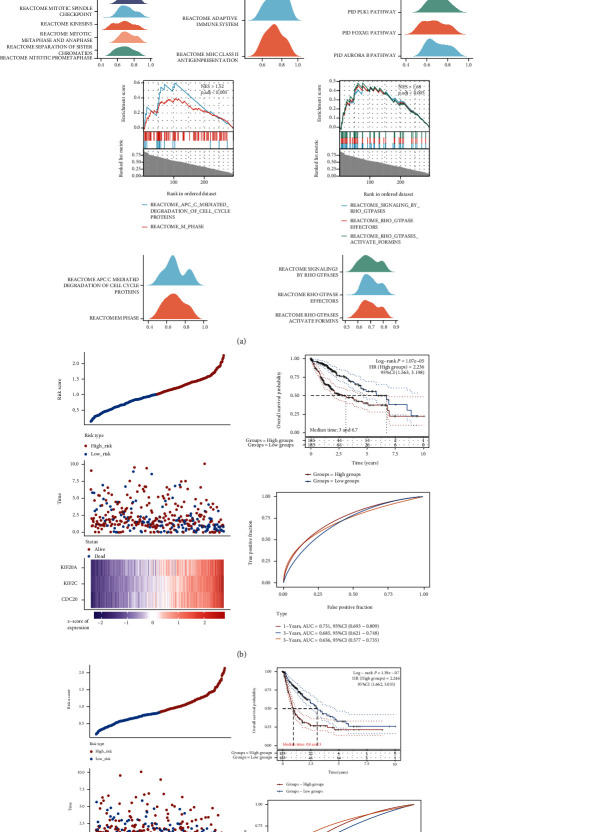
GSEA analysis of PRC1 co-expressing genes and the prognostic signature of immune system-related genes. (a) Identification of enriched gene sets by GSEA analysis: kinesins; immune system; PLK1, FOXM1, and Aurora B pathway; cell cycle; and RHO GTPase. OS analysis (b) and PFS analysis (c) of the gene signatures which were associated with the immune system according to the GSEA analysis. The left panel showed the curve of risk score, survival status of the patients, and the heat map of the expression profiles of the prognostic genes in the low- and high-risk group. The right upper panel showed the Kaplan-Meier survival analysis of the gene signature. The right lower panel showed the time-dependent ROC analysis of the gene signature.

To further explore the functions of PRC1 in LIHC, GSEA analysis of the top 300 PRC1-related genes was conducted. GSEA analysis showed that PRC1 regulated the cell mitosis and kinesins, which were in accordance with previous studies (Figure 7(a)). PRC1 was predicted to affect the PLK1, FOXM1, and Aurora B pathways, which were all essential cell cycle regulating pathways (Figure 7(a)). Moreover, RHO GTPases, the actin reorganization controllers affecting cell motility, were also enriched (Figure 7(a)). It is noteworthy that PRC1 was associated with the adaptive immune system and MHC II antigen presentation, which were unreported new functions (Figure 7(a)). To investigate the importance of these immune-regulating genes, we build the signatures of PRC1 coexpressing immune-related genes and tested their prognostic values using data from TCGA database. The signatures of immune-related genes showed promising prognostic value in LIHC. Among the immune-related genes coexpressed with PRC1, high expressions of KIF20A, KIF2C, and CDC20 predicted worse OS (median time: 3 VS 6.7 years) (Figure 7(b)). Likewise, RACGAP1, KIF20A, KIF20A, KIF18A, KIF4A, CENPE, KIF2C, and UBE2C also formed a signature, the high expression of which predicted worse PFS (median time: 0.9 VS 3 years) (Figure 7(c)). These results indicate that the immune-regulating functions of PRC1 may be important in influencing the prognosis of LIHC.

### 3.7. PRC1 Correlates with the Immune-Suppressive Microenvironment

**Figure 8:**
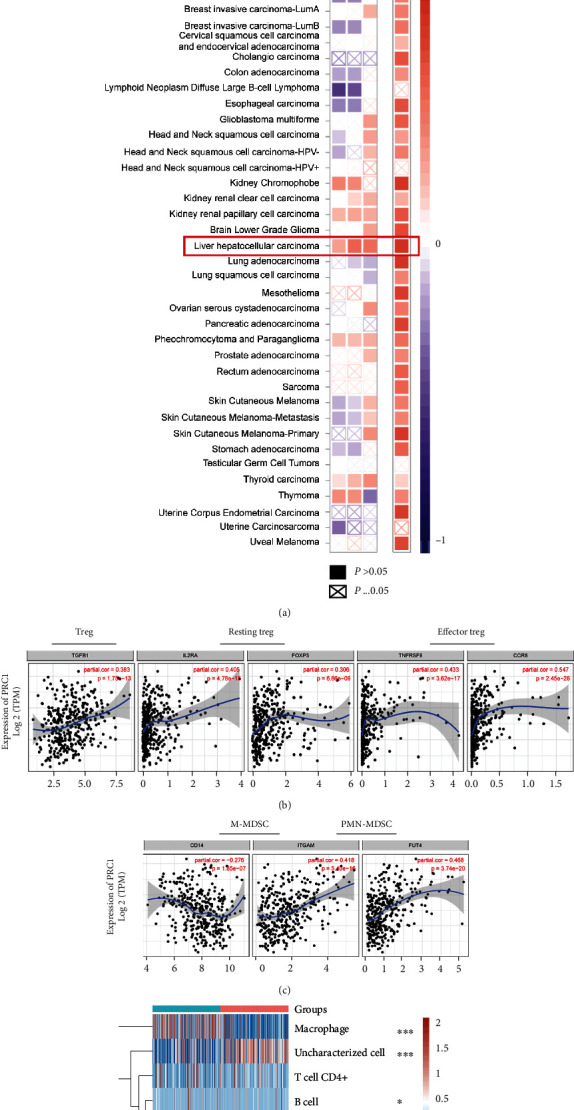
PRC1 overexpression was positively correlated with suppressive immune cells' infiltration and negatively related with effector immune cells' infiltration. (a) Pan-cancer correlation analysis of the PRC1 expression and the infiltration of Treg and MDSC cells. (b–c) Correlation of the expression of PRC1 and the marker genes of Treg and MDSC in LIHC. (d) The association between the PRC1 expression and the macrophage, B cell, CD4+ and CD8+ T cell, NK cell, and endothelial cell. ∗*p* < 0.05; ∗∗∗*p* < 0.001.

To learn more about the immune-regulating functions of PRC1, we analyzed the correlation between PRC1 expression and immune cells' infiltration. The results of CIBERSORT, CIBERSORT-Abs, and QUANTISEQ analyzing strategies all showed that PRC1 expression had a significant positive correlation with the infiltration of Treg cells in LIHC and other cancers including KIRC, KIRP, PCPG, and THCA (Figure 8(a)). TIDE analysis demonstrated the correlation between PRC1 expression and MDSC infiltration in pan-cancer fields, especially in LIHC (Figure 8(a)). To further validate the association of PRC1 with suppressive immune microenvironment, we evaluated the correlation of PRC1 with the expression of biomarkers of Tregs. PRC1 was positively related with all 5 markers of both effector Treg and resting Treg cells (Figure 8(b)). We also analyzed the expression of the gene markers of polymorphonuclear-/monocytic-MDSCs (PMN-/M-MDSCs). Interestingly, the PRC1 expression was positively related with the PMN-MDSC cell marker, while negatively related with the marker of M-MDSC cells, indicating a possible differentiation selection function of PRC1 (Figure 8(c)). Moreover, the infiltrations of the effector immune cells including B cells and CD8+ T cells were negatively related with PRC1 expression, according to the bulk RNA sequencing data of LIHC cases from TCGA database (Figure 8(d)).

Inflammatory cytokines in TME play important roles in cancer progression, invasion, and metastasis. The correlation between PRC1 and cytokines was analyzed and adjusted for tumor purity. PRC1 are positively associated with the expression of inflammatory cytokine genes, including the IL family (IL1B, IL2, IL6, IL10, IL17A, and IL18); CCL1, 5, 17, 18, 20, 22, and 28; CXCL10; CCR4, 5, 8, and 10; VEGFA; TNFRSF6B; TGFB1; and TNF (Table 2).

### 3.8. The Ability of PRC1 Predicting LIHC Patients' Prognosis Relies on the T-reg Cell Abundance

**Figure 9:**
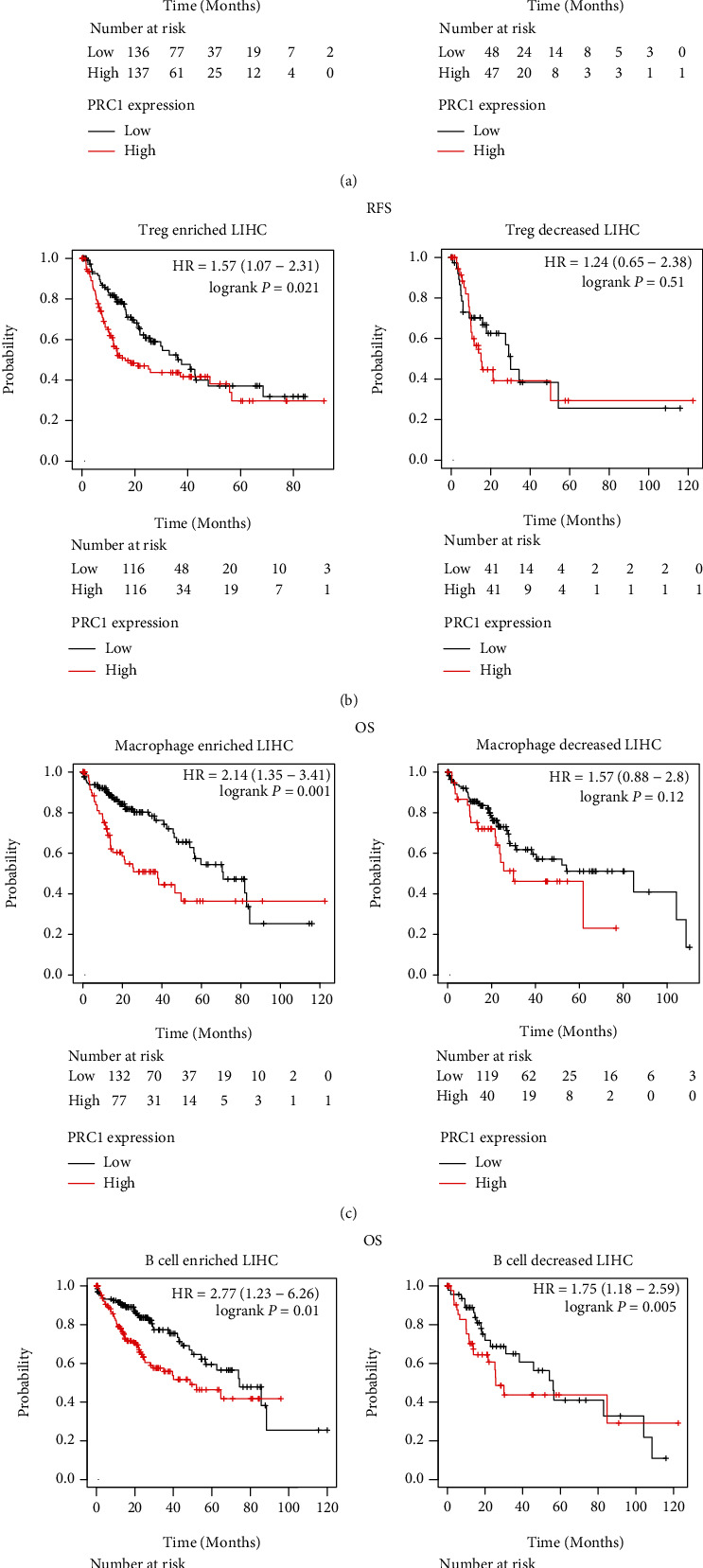
Survival curves according to high and low expression of PRC1 in LIHC grouped by Treg, macrophage, or B cell infiltration. Correlation of PRC1 expression and OS (a) and RFS (b) in Treg-enriched and decreased LIHC subgroups were estimated by Kaplan-Meier plotter. OS analyses of PRC1 in LIHC cases grouped by macrophage (c) or B cell (d) infiltration.

Given the close connection of PRC1 and the immune system, we wondered the contribution of suppressive immune cells in PRC1 impacting LIHC prognosis. Thus, we analyzed the OS and RFS of high and low PRC1 expression subgroup in Treg-enriched and decreased LIHC patients in the TCGA database. We found that in Treg-enriched cases, high PRC1 expression was profoundly related with poor OS and RFS (Figures 9(a) and 9(b)). The median survival time for Treg-enriched LIHC patients with high PRC1 expression (divided by median expression) was 46.57 months, while 71.03 months for low PRC1 expression patients. However, in Treg-decreased LIHC cases, the OS and RFS showed no difference between high and low PRC1 expression subgroup (Figures 9(a) and 9(b)). Similarly, the relative absence of total macrophage in LIHC abolished the association between PRC1 expression and OS (Figure 9(c)). Nevertheless, the enrichment or decrease of B cells did not influence the prognostic value of PRC1 in LIHC (Figure 9(d)). These results indicate that the prognostic value of PRC1 relies on regulating Treg and total macrophage infiltration.

### 3.9. PRC1 Associates with the Expression of Immune Checkpoint Marker Genes

**Figure 10:**
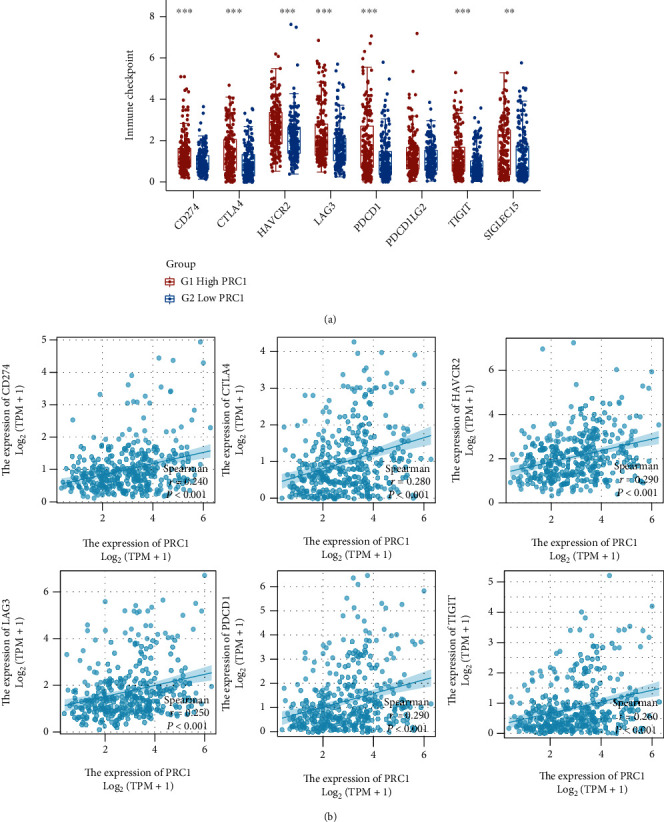
Correlation of PRC1 expression with immune checkpoint molecules. (a) The expressions of immune checkpoint molecules were significantly higher in the LIHC group with high PRC1 expression. (b) The expressions of immune checkpoint molecules were significantly associated with PRC1. ∗∗*p* < 0.01; ∗∗∗*p* < 0.001.

Upregulated immune checkpoints or their ligands within the tumor microenvironment are common inhibitory mechanisms for LIHC to evade antitumor immunity. We investigated the expression of immune checkpoint gene markers in LIHC groups separated by high or low PRC1 expression. The expression of CD274 (PD-L1), CTLA4, HAVCR2, LAG3, PDCD1, TIGHT, and SIGLEC15 was significantly higher in LIHC samples with high PRC1 expression (Figure 10(a)). Consistently, these immune checkpoint genes were closely correlated with PRC1 (Figure 10(b)).

## 4. Discussion

PRC1 safeguards cell division process by regulating microtubule binding and central spindle assembly [31]. Recent studies found that it might also play a role in tumorigenesis. Knockdown or knockout of PRC1 inhibited the growth, metastasis, recurrence, and/or drug resistance of various cancers [32, 34, 35, 63]. The underlying mechanisms have not been fully understood. Chen et al. firstly found the PRC1 overexpression in LIHC by digging their microarray data [30]. They focused on the transcriptional regulators of PRC1, by analyzing the binding site on PRC1 promoter region. And their work well explained the positive feedback loop between PRC1 and the Wnt/*β*-catenin pathway in LIHC. However, the molecule functions of PRC1 need further depiction. We conducted bioinformatic function analyses to fully depict the function atlas of the overexpressed PRC1 in LIHC. Notably, our work firstly identified the immune suppression function of PRC1 in LIHC. Upregulated PRC1 was associated with more advanced clinical stages and predicted poor prognosis of LIHC. GO and KEGG analyses of the coexpressing genes of PRC1 revealed that PRC1 regulated the process of mitosis, HR, and DDR. Consistently, PRC1 was positively correlated with the tumor genomic instability biomarkers, MSI, HRD, and LOH. Moreover, GSEA analysis revealed that PRC1 might regulate TME. By further investigation, we found that PRC1 was positively correlated with the infiltration of immunosuppressive cells, Treg, and MDSC. The control of Treg infiltration might be the way of PRC1 exerting influence on LIHC prognosis. High PRC1 expression was also related with the expression of immune checkpoint molecules in LIHC. These findings suggest that PRC1 may be a prognostic biomarker and a target for immunotherapy.

Cancers with higher mutation burden or genomic instability have more potential neoantigens [64, 65], and are thus more sensitive to ICI therapy or DDR molecule inhibitors like poly ADP-ribose polymerase (PARP) inhibitor [66, 67]. Thus, TMB, MSI, and PD-L1 have become important biomarkers for immunotherapy, the complimentary utilization of which has the potential to predict ICIs responsiveness better than each alone [60]. Our work found that PRC1 was positively related with the MSI score and PD-L1 expression of LIHC, indicating their internal interactions. The relationship between TMB, MSI, and PD-L1 has been explored broadly, but the overlap in cancers among which is rare [68]. Moreover, 69.5% of all cancer cases were negative for all 3 biomarkers [69]. Thus, these 3 biomarkers may not be sufficient in the utilization of personalized therapy response prediction. HR is an important part of the DDR process, responsible for the reparation of double-strand breaks (DSBs) [70]. HRD is an emerging biomarker defined by the mutations of BRCA1/2 genes, along with other Fanconi anemia pathway genes (RAD51D, NBN, and ATM) [71, 72]. Currently, HRD has not been used in clinical tests, but it has showed profound ability in predicting patients' response to platinum-based chemotherapies and cytotoxic agents that cause DNA damage [73, 74]. Our work found the high correlation between PRC1 and the HRD of LIHC for the first time. Consistently, function analysis in our study also revealed the possible regulation of the Fanconi anemia pathway by PRC1. LOH is an indicator of homologous repair deficiency and is commonly associated with the inactivation of tumor suppressor genes [75]. The two major mechanisms inducing LOH are deletion of chromosomal fragments and mitotic recombination between homologous alleles [76]. As a mitosis-regulating protein, PRC1 is essential for the chromosome dynamic regulation [36]. Our work first demonstrated the positive correlation between PRC1 and LOH in LIHC. Taken together, PRC1 has great potential in predicting patients' responses to ICI therapy and DDR inhibitors.

PRC1 may hold functions far more than we have noticed. GSEA analysis of the PRC1 coexpressing genes found it involved in the RHO GTPase signaling. RHOA is a small GTPase protein in the RHO GTPases family. Previous studies in gastric cancer found that RHOA Y42 mutation in cancer cells produced excessive fatty acids by upregulating fatty acid synthase (FASN) [77]. The fatty acids produced by tumor cells and stromal cells in the TME can be utilized by Tregs as an energy source for their survival and immune suppressive function [78]. As a result, the TME rich in fatty acid recruits Tregs while expelling CD8+ T cells. This mechanism contributes to the impaired sensitivity to PD-1 targeting immunotherapy [77]. Since the RHOA mutation can lead to the fatty acids production in TME by gastric tumor cells, we speculate that in the TME of LIHC, maybethe upregulated PRC1 recruits Tregs by regulating RHOA and its RHO family.

The TME of LIHC is abundant in immunosuppressive cells and secreted molecules, which contributes to the resistance to immunotherapy [79]. Abnormal gene expression can promote tumor cells to produce chemokines, which helps to recruit Tregs to the TME. Tregs have multiple chemokine receptors, such as CCR4-CCL17/22, CCR5-CCL5, CCR8-CCL1, and CCR10-CCL28. We found that PRC1 was positively related to the gene expressions of multiple chemokines and receptors like CCL1, 5, 17, 18, 20, 22, and 28; CXCL10; and CCR4, 5, 8, and 10. The increased secretion of chemokines and increased expression of corresponding receptors following the PRC1 upregulation may contribute to the Treg enrichment in TME.

Other than shaping the TME, we found that PRC1 was associated with the PLK1 pathway, which was in accordance with previous studies in Ewing sarcoma (EwS) [80]. High expression of PRC1 in EwS implied a better response to volasertib, a PLK1 inhibitor [80]. PLK1 activates PRC1, forming the PRC1-PLK1 protein complex and helping PRC1 translocate to the central spindle to initiate cytokinesis. However, PLK1 can also negatively regulate PRC1 to prevent premature midzone formation before cytokinesis [81]. The negative regulation of PRC1 by PLK1 is initiated by microtubules, creating a potential negative feedback loop controlling PRC1 activity. PLK1 is found overexpressed in most cancers and is associated with a poor prognosis. By now, PLK1 inhibitors are undergoing clinical trials of various cancers. PRC1 expression may be a potential marker to predict the antitumor efficacy of PLK1 inhibitors.

Our study depicted the correlation between PRC1 overexpression and the immune-suppressive TME in LIHC. To our knowledge, this is the first study to explore the immune-regulatory function of PRC1. Nevertheless, the limitation of our study lies in the unexplored precise pathway through which PRC1 regulates the immune system. In the subsequent research, we will further investigate the underlying mechanisms of PRC1 regulating the TME of LIHC. Taken together, we propose PRC1 as a biomarker and a target for the immunotherapy of LIHC.

## Figures and Tables

**Table 1:** The baseline characteristics of the LIHC patients from the TCGA database.

Characteristic	Low expression of PRC1	High expression of PRC1	*p*
n	187	187	
Age, *n* (%)			0.006
≤60	75 (20.1%)	102 (27.3%)	
>60	112 (30%)	84 (22.5%)	
Gender, *n* (%)			0.377
Female	56 (15%)	65 (17.4%)	
Male	131 (35%)	122 (32.6%)	
Race, *n* (%)			0.044
Asian	66 (18.2%)	94 (26%)	
Black or African American	9 (2.5%)	8 (2.2%)	
White	101 (27.9%)	84 (23.2%)	
Tumor status, *n* (%)			0.005
Tumor free	115 (32.4%)	87 (24.5%)	
With tumor	63 (17.7%)	90 (25.4%)	
T stage, *n* (%)			0.017
T1	106 (28.6%)	77 (20.8%)	
T2	41 (11.1%)	54 (14.6%)	
T3	32 (8.6%)	48 (12.9%)	
T4	5 (1.3%)	8 (2.2%)	
N stage, *n* (%)			0.624
N0	122 (47.3%)	132 (51.2%)	
N1	1 (0.4%)	3 (1.2%)	
M stage, *n* (%)			0.361
M0	130 (47.8%)	138 (50.7%)	
M1	3 (1.1%)	1 (0.4%)	
Pathologic stage, *n* (%)			0.009
Stage I	99 (28.3%)	74 (21.1%)	
Stage II	40 (11.4%)	47 (13.4%)	
Stage III	32 (9.1%)	53 (15.1%)	
Stage IV	4 (1.1%)	1 (0.3%)	
BMI, *n* (%)			0.207
≤25	83 (24.6%)	94 (27.9%)	
>25	87 (25.8%)	73 (21.7%)	
AFP(ng/ml), *n* (%)			< 0.001
≤400	126 (45%)	89 (31.8%)	
>400	19 (6.8%)	46 (16.4%)	
Child-Pugh grade, *n* (%)			1.000
A	122 (50.6%)	97 (40.2%)	
B	12 (5%)	9 (3.7%)	
C	1 (0.4%)	0 (0%)	
Age, median (IQR)	64 (54.5, 69.5)	59 (50.25, 67.75)	0.005

**Table 2:** Correlation analysis between PRC1 and cytokines in LIHC.

Cytokines	None		Purity	
	Cor	*p*	Cor	*p*
IL1B	0.318	∗∗∗	0.424	∗∗∗
IL2	0.070	0.192	0.118	∗
IL6	0.053	0.325	0.162	∗∗
IL10	0.221	∗∗∗	0.359	∗∗∗
IL17A	0.099	0.066	0.109	∗
IL18	0.205	∗∗∗	0.371	∗∗∗
TNFRSF6B	0.319	∗∗∗	0.354	∗∗∗
TGFB1	0.265	∗∗∗	0.383	∗∗∗
TNF	0.267	∗∗∗	0.395	∗∗∗
VEGFA	0.581	∗∗∗	0.564	∗∗∗
CXCL10	0.234	∗∗∗	0.278	∗∗∗
CCL1	0.122	∗	0.1538	∗∗
CCL5	0.114	∗	0.235	∗∗∗
CCL17	0.057	0.283	0.131	∗
CCL18	0.083	∗	0.179	∗∗∗
CCL20	0.258	∗∗∗	0.297	∗∗∗
CCL22	0.106	∗	0.249	∗∗∗
CCL28	0.360	∗∗∗	0.347	∗∗∗
CCR4	0.266	∗∗∗	0.383	∗∗∗
CCR5	0.250	∗∗∗	0.423	∗∗∗
CCR8	0.447	∗∗∗	0.547	∗∗∗
CCR10	0.464	∗∗∗	0.495	∗∗∗

∗*p* < 0.05, ∗∗*p* < 0.01, ∗∗∗*p* < 0.001.

## Data Availability

The original contributions presented in the study are included in the article/Supplementary Material. Further inquiries can be directed to the corresponding author.

## Abbreviations

ACC: Adrenocortical carcinoma
BLCA: Bladder urothelial carcinoma
BRCA: Breast invasive carcinoma
CESC: Cervical squamous cell carcinoma and endocervical adenocarcinoma
CHOL: Cholangiocarcinoma
COAD: Colon adenocarcinoma
DLBC: Lymphoid neoplasm diffuses large B cell lymphoma
STES: Stomach and esophageal carcinoma
GBM: Glioblastoma multiforme
HNSC: Head and neck squamous cell carcinoma
KICH: Kidney chromophobe
KIRC: Kidney renal clear cell carcinoma
KIRP: Kidney renal papillary cell carcinoma
LAML: Acute myeloid leukemia
LGG: Brain lower grade glioma
LIHC: Liver hepatocellular carcinoma
LUAD: Lung adenocarcinoma
LUSC: Lung squamous cell carcinoma
MESO: Mesothelioma
OV: Ovarian serous cystadenocarcinoma
PAAD or PADD: Pancreatic adenocarcinoma
PCPG: Pheochromocytoma and paraganglioma
PRAD: Prostate adenocarcinoma
READ: Rectum adenocarcinoma
SARC: Sarcoma
SKCM: Skin cutaneous melanoma
STAD: Stomach adenocarcinoma
TGCT: Testicular germ cell tumors
THYM: Thymoma
THCA: Thyroid carcinoma
UCS: Uterine carcinosarcoma
UCEC: Uterine corpus endometrial carcinoma
UVM: Uveal melanoma
LIHC: Liver hepatocellular carcinoma
PRC1: Protein regulator of cytokinesis 1
TCGA: The Cancer Genome Atlas
GEO: Gene Expression Omnibus
GO: Gene Ontology
KEGG: Kyoto Encyclopedia of Genes and Genomes
GSEA: Gene Set Enrichment Analysis; Tregs, T regulatory cells
PMN-MDSCs: Polymorphonuclear myeloid-derived suppressor cells
ICIs: Immune checkpoint inhibitors
PD-1/PD-L1: Programmed cell death/-ligand 1
TME: Tumor microenvironment
TAMs: Tumor-associated macrophages
FoxP3: Forkhead box protein P3
Teff: T effector cell
CTLA4: Cytotoxic T lymphocyte-associated antigen 4
LAG3: Lymphocyte-activation gene 3
TIM3: T cell immunoglobulin and mucin domain containing-3
VEGF: Vascular endothelial growth factor
TGF-*β*: Transforming growth factor*-β*
IL-10: Interleukin-10
IMAEs: Immune-mediated adverse events
CDKs: Cyclin-dependent kinases
PLK1: Polo-like kinase 1
DEGs: Differentially expressed genes
GDC: The Genomic Data Commons data portal
IHC: Immunohistochemistry
HPA: Human Protein Atlas
OS: Overall survival
PFI: Progression-free interval
DSS: Disease-specific survival
RFS: Recurrence-free survival
MF: Molecular function
CC: Cellular components
BP: Biological processes
TMB: Tumor mutation burden
MSI: Microsatellite instability
LOH: Loss of heterozygosity
HRD: Homologous recombination deficiency
PFS: Progression-free survival
HR: Homologous recombination
FA: Fanconi anemia
PARP: Poly ADP-ribose polymerase inhibitors
CIN: Chromosomal instability
DSBs: Double-strand breaks
APC: Antigen presenting cell.

## Supplementary Materials

Supplementary Materials Supplementary Table 1. The sample characteristic statistics of GSE84402 dataset. Supplementary Table 2. The expression of the top 300 PRC1 coexpressed genes in LIHC dataset in TCGA database. Supplementary Figure 1. The pan-cancer prognostic value of PRC1.
