# Supplementary material for: Protein Regulator of Cytokinesis 1 (PRC1) Upregulation Promotes Immune Suppression in Liver Hepatocellular Carcinoma
Source: J Immunol Res. 2022 Aug 9;2022:7073472. doi: 10.1155/2022/7073472 (PMC9381293; doi:10.1155/2022/7073472)
Supplement: Supplementary Materials — Supplementary Table 1. The sample characteristic statistics of GSE84402 dataset. Supplementary Table 2. The expression of the top 300 PRC1 coexpressed genes in LIHC dataset in TCGA database. Supplementary Figure 1. The pan-cancer prognostic value of PRC1. [file 7073472.f1.pdf]

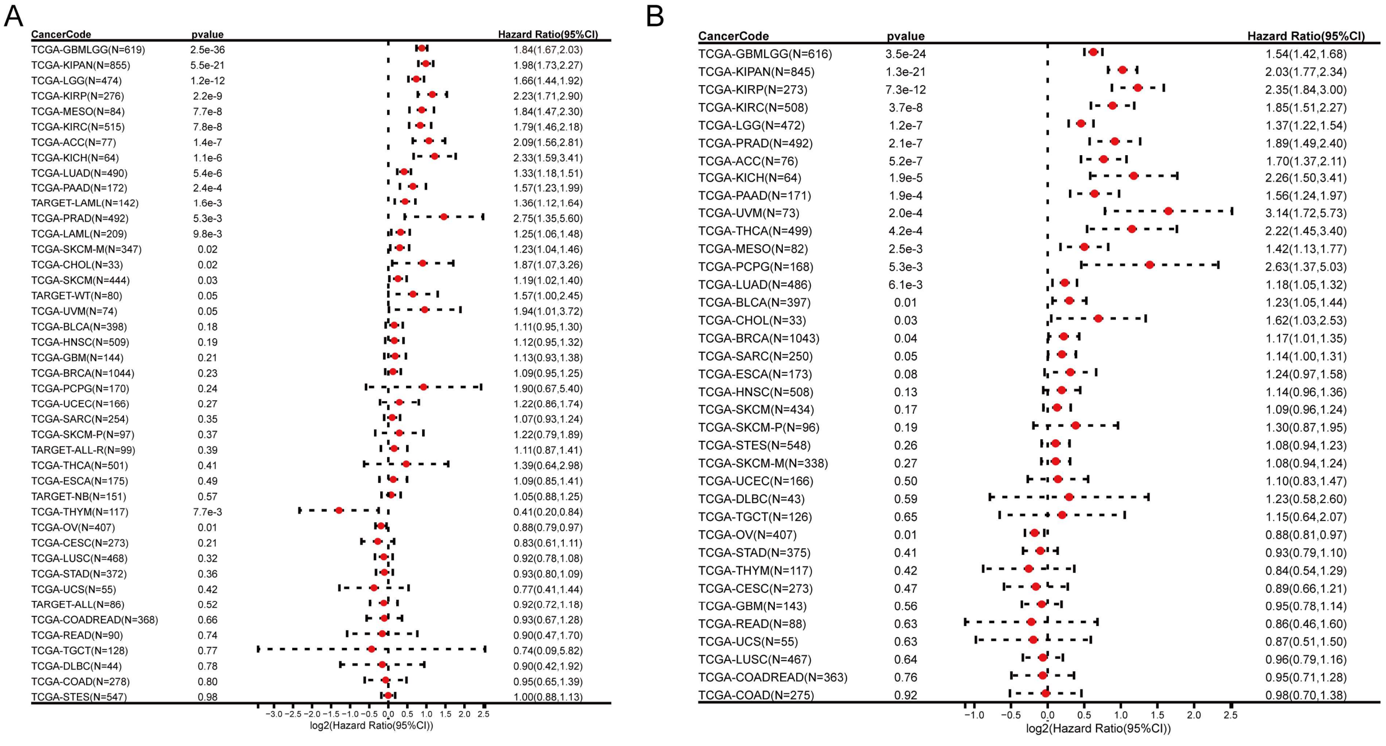

Supplementary Figure 1. The pan-cancer prognostic value of PRC1

A. A forest diagram shows the pan-cancer correlation between PRC1 expression and OS.

B. A forest diagram shows the pan-cancer correlation between PRC1 expression and PFI.

Supplementary Table 1. The sample characteristic statistics of GSE84402 dataset

Characteristic	cancertissue	non-canceroustissue	p
n	14	14	
hbsag, n (%)			1.000
negative	1 (3.6%)	1 (3.6%)	
positive	13 (46.4%)	13 (46.4%)	
gender, n (%)			1.000
female	5 (17.9%)	5 (17.9%)	
male	9 (32.1%)	9 (32.1%)	
age, mean ± SD	47.69 ± 9.92	47.69 ± 9.92	1.000

Supplementary Table 2. The expression of the top 300 PRC1 co-expressed genes in LIHC dataset in TCGA database

gene	Log2FC
BUB1B	0.684333368
KIF23	0.859827215
CKAP2L	0.662927038
ARHGAP11A	0.530113597
NUSAP1	0.452746956
TOP2A	0.758353631
KIF18B	0.665194448
FANCI	0.486302827
ANLN	0.924572728
FOXM1	0.547705404
KIF18A	0.741723356
BUB1	0.752744257
TPX2	0.716879792
KIF11	0.705653533
KIFC1	0.643708728
POLQ	0.652941671
KIF4A	0.747388251
TICRR	0.593424262
E2F8	0.369028049
NCAPH	0.743188771
SGO2	0.671210601
OIP5	0.41277369
CENPF	0.68165795
CDCA8	0.641631396
NCAPG	0.61302592
CENPI	0.712138895
CDK1	0.699164631
HJURP	0.703444779
GTSE1	0.715364772
MELK	0.640516629
CENPE	0.762804349
MCM10	0.71600505
RAD51AP1	0.450228624
CENPK	0.427419683
STIL	0.551929804
FANCD2	0.49830215
NEK2	0.617518664
ERCC6L	0.753608235
GINS1	0.542130598
CCNB2	0.566808874
ECT2	0.609155174
PLK4	0.589155238
KIF20A	0.666342435
DLGAP5	0.842234397
MKI67	0.714459478
SGO1	0.603345374
NUF2	0.713201875
CIP2A	0.729293151
RAD54L	0.809560913
LMNB1	0.388404628
PARPBP	0.40776461
TTK	0.699862747
XRCC2	0.453887448
UHRF1	0.698796057
GPSM2	0.623014156
KIF14	0.700107334
EXO1	0.705120252
KIF15	0.631061966
PLK1	0.829446028
SPDL1	0.318724888

gene	Log2FC
ZWINT	0.513144342
CCNA2	0.194279504
RACGAP1	0.600337428
WDHD1	0.557225073
AC099850.3	0.78811344
DEPDC1	0.762905353
TCF19	0.456979877
CENPL	0.539285211
ATAD5	0.434474133
AC091057.1	0.475941016
DTL	0.475850981
HELLS	0.519462748
SKA3	0.440290342
NDC80	0.603510129
EZH2	0.41750497
PRR11	0.861723505
NCAPG2	0.382036237
CDCA2	0.819871047
MCM2	0.49747493
ASF1B	0.634997052
KNTC1	0.511397195
WDR76	0.381611643
HASPIN	0.510929925
MAD2L1	0.656280951
EME1	0.5193216
ASPM	0.620829091
TRIP13	0.667102073
CEP55	0.978111473
CDC6	0.741029374
DEPDC1B	0.835381528
ZWILCH	0.18794166
SPC25	0.539333113
FAM72B	0.580196376
RRM2	0.462637565
CDCA5	0.592500023
TROAP	0.703322675
HMMR	0.468944271
RAD51	0.484322594
WDR62	0.636712163
PBK	0.612972775
CCNB1	0.53277189
KIF2C	0.850629865
CDC45	0.587820737
CENPA	0.82550885
CCNF	0.517905327
CLSPN	0.735642476
SKA1	0.834921388
C5orf34	0.410339336
RBL1	0.512606308
BARD1	0.572677153
BRCA1	0.381392881
KIF20B	0.343531518
ORC1	0.616479031
ORC6	0.638443613
PCLAF	0.549284804
E2F2	0.708964857
CDCA3	0.562907112
PSMC3IP	0.362717635
HROB	0.365176912
PIF1	0.522665609

Supplementary Table 2. The expression of the top 300 PRC1 co-expressed genes in LIHC dataset in TCGA database

gene	Log2FC
DBF4B	0.312861993
ESCO2	0.59095814
FAM72A	0.489266959
NCAPD2	0.474152866
MCM6	0.440892003
DNMT1	0.320508855
KNSTRN	0.162258725
MMS22L	0.42630918
CHAF1B	0.705531467
PKMYT1	0.638191771
SHCBP1	0.905898567
HMGB2	0.324668771
FANCB	0.538410002
GAS2L3	0.689716393
MCM8	0.3797564
CIT	0.549822453
FANCG	0.289033339
TACC3	0.54729995
BRCA2	0.332222789
AC112777.1	0.792726575
CDC25C	0.443969961
BIRC5	0.759817598
ARHGEF39	0.428619846
CHEK1	0.450443085
KPNA2	0.447695464
NEMP1	0.331128778
MYO19	0.306509133
MCM3	0.338981505
CDKN3	0.404920472
RFC4	0.354407536
LIG1	0.221009286
MYBL2	0.820969949
KIF24	0.506677283
CENPH	0.355390148
SPAG5	0.352220162
MCM4	0.273079731
CHAF1A	0.199541335
BLM	0.435922861
C21orf58	0.330795032
INCENP	0.306175197
NSD2	0.229563681
DBF4	0.323179365
CDT1	0.462349172
RFC5	0.170079098
UBE2C	0.860527665
MCM5	0.232023914
C4orf46	0.40510741
DSN1	0.176314324
CDC25A	0.522759413
CDC20	0.853128835
GINS4	0.412340466
CBX1	0.240493363
MTFR2	0.612737333
STMN1	0.399717996
UBE2T	0.490494804
CENPO	0.48005077
CDK2	0.263741621

gene	Log2FC
TOPBP1	0.256128487
DONSON	0.160810857
TIMELESS	0.170146779
TYMS	0.488545452
EMC3-AS1	0.489691484
POLA1	0.396159007
FBXO43	0.222177667
DNAJC9	0.124935469
E2F1	0.486651945
MSH2	0.245395539
FAM111B	0.328591578
FEN1	0.255979766
MCM7	0.447840648
CENPU	0.485627941
LIN9	0.393337312
MTBP	0.2538593
POLD1	0.315802104
CDCA4	0.40559456
ESPL1	0.525307336
CKAP5	0.141167456
CEP152	0.333432933
PRIM2	0.213199372
SPATS2	0.343551221
MASTL	0.256974232
MIS18A	0.225303952
DDIAS	0.495990458
CDC7	0.464400293
DIAPH3	0.110007735
RRM1	0.195258786
TUBA1B	0.353294984
PHF19	0.505145027
RHNO1	0.254681653
AURKB	0.666968819
SMC4	0.39934797
TRIM59	0.540154988
CENPM	0.677701964
KIAA1841	0.340377372
ILF3	0.150327495
NUP107	0.210654255
CPSF6	0.203906588
E2F7	0.734645372
NUP155	0.156234556
CCNE2	0.563784259
FBXO5	0.546071296
SASS6	0.316272537
NDC1	0.281008653
RAD1	0.121656505
CKAP2	0.327110238
SMPD4	0.122767118
CENPQ	0.286966296
XRCC3	0.173742995
GINS3	0.383538974
SUZ12	0.151560983
PCNA	0.28385525
ACTL6A	0.197693145
SMC2	0.379873349
AURKA	0.130931264
RTKN2	0.503678767
TRAIP	0.530491055
ZNF207	0.13051535

Supplementary Table 2. The expression of the top 300 PRC1 co-expressed genes in LIHC dataset in TCGA database

gene	Log2FC
RAD18	0.209832774
MGME1	0.087412552
TMPO	0.052291687
CASP2	0.227390702
NUP205	0.231517871
BRIP1	0.30006232
DDX12P	0.255162991
HNRNPL	0.094348352
DNA2	0.299160714
GMPS	0.166080593
USP1	0.348903215
NEDD1	0.241116893
SLC25A19	0.280724894
TEDC2	0.521287938
ANKLE2	0.135860882
SUV39H2	0.188547429
CEP250	0.16231527
HNRNPA3	0.086087087
H2AZ1	0.324909617
RMI2	0.510035388
RNF34	0.171478287
TTF2	0.283476602
SMARCD1	0.165622024
NUP85	0.224538612
HSPA14	0.187164069
CBFA2T2	0.147757081
NEIL3	0.75511675
NDE1	0.317495984
RFC3	0.13637812
LMNB2	0.595731941
SSRP1	0.137094717
CEP78	0.212247915
DCLRE1B	0.274548949
RFWD3	0.238032072
ARHGAP33	0.365949268
PTTG1	0.667542427
RAD51D	0.267616837
MYBL1	0.402312084
TUBG1	0.186692019
TMPO-AS1	0.083050322
BORA	0.276015672
AUNIP	0.547065961
CENPJ	0.359279181
MAPKAPK5	0.077530171
SLBP	0.119680496
ZNF724	0.311972803
SRRT	0.12913508
KPNB1	0.233628062
NRM	0.577063029
U2SURP	0.087106183
COMMD2	0.057439966
PRKCI	0.183288954
TTI1	0.087460276
SRSF2	0.062384168
ZGRF1	0.340022672
SENP1	0.149003245
FANCE	0.404489732
POLD3	0.200412941
VRK1	0.170551953
MACROH2A1	0.13858448
PPHLN1	0.09484003
SART3	0.098679921
USP39	0.127652446
